# Supplementary material for: Vibronic coherence contributes to photocurrent generation in organic semiconductor heterojunction diodes
Source: Nat Commun. 2020 Jan 30;11:617. doi: 10.1038/s41467-020-14476-w (PMC6992633; doi:10.1038/s41467-020-14476-w)
Supplement: Supplementary file 1 — Supplementary Information [file 41467_2020_14476_MOESM1_ESM.docx]

Vibronic coherence contributes to photocurrent generation in organic semiconductor heterojunction diodes

Bian et al.

**Supplementary Figures**

**a**

**b** **c**

**d e**

**Supplementary Figure 1.** Photovoltaic performances and EQE profile of devices. **a.** Absorption and photoluminescence for pristine materials. **b.** PCDTBT ratio dependent device performance. It is obvious optimized ratio for D1:D2:A1 (PTB7-th:PCDTBT:PC_71_BM) is 9:1:10 and 7:3:10. **c.** Current density-voltage (*J-V*) characteristics of binary and ternary devices under 1 sun illumination and dark. **d.** EQE spectra of binary and ternary devices. **e.** The photovoltaic results (*PCE*) of all binary and ternary devices. In both three blends, the data shows the maximum, the minimum and the average PCE. For ternary blends, the third component PCDTBT with different molecular weight (Supplementary Note 1).

**Supplementary Figures**

**a**

**b**


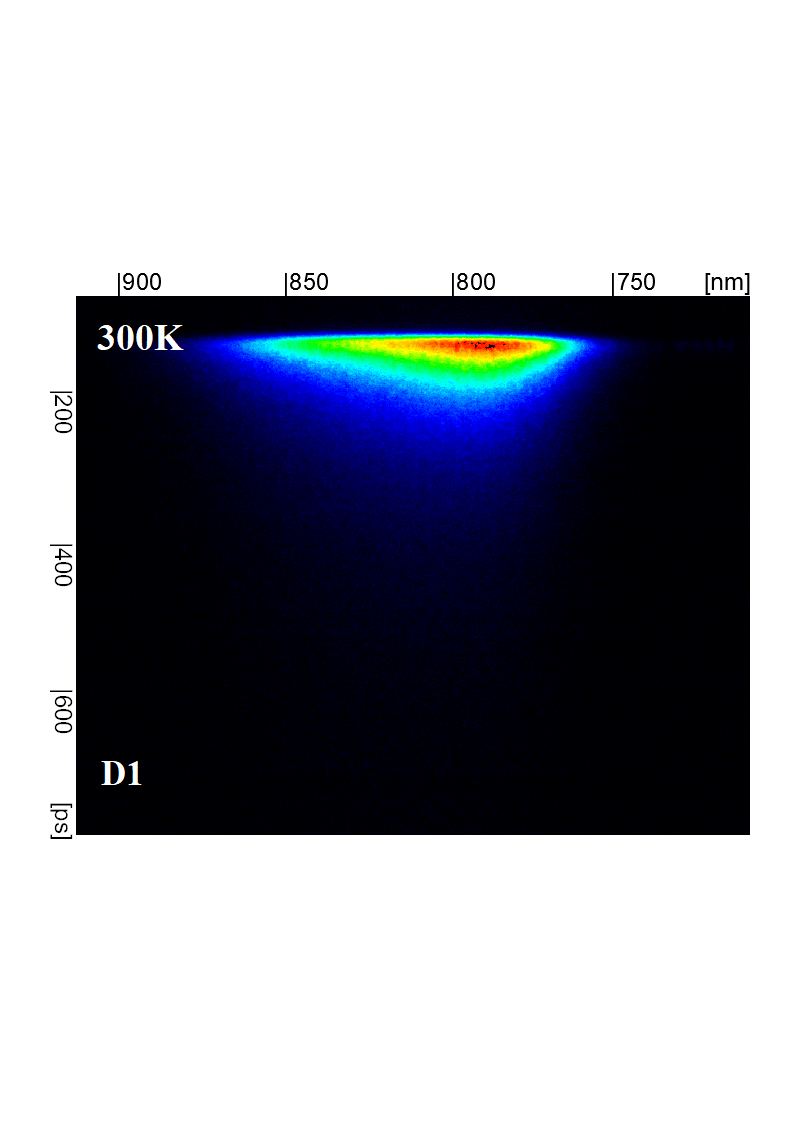
 **
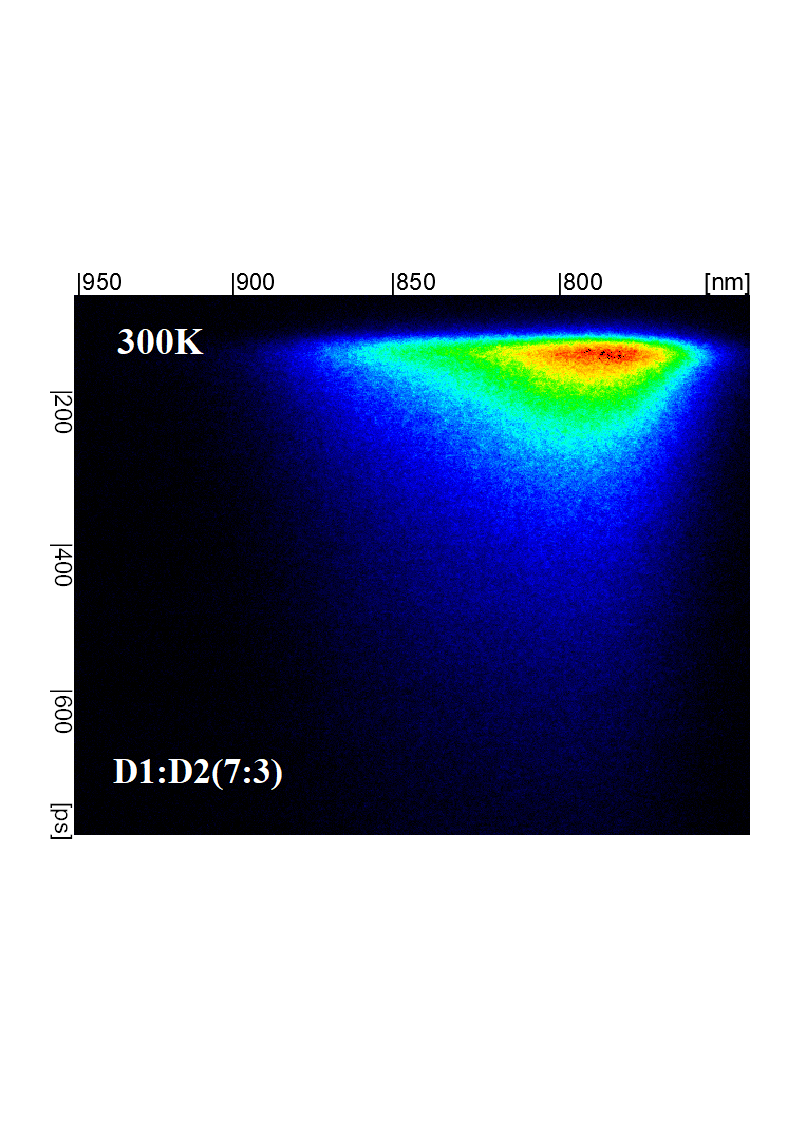
**

**Supplementary Figure 2.** Excited-state dynamics of pristine material and two donor blends. **a**. Transient absorption spectra of pristine D1 and D1:D2 (7:3) blends, pump at 550nm. **b**. Time-resolved photoluminescence of pristine D1 and D1:D2 (7:3) blends, pump at 700nm. Energy transfer between D1 and D2 discussed in the Supplementary Note 2.

**Supplementary Figures**

**a**  **b**

**c**  **d**

**Supplementary Figure 3.** Excited-state dynamics of blends. Transient absorption spectra of two binary blends ( (**a)** D_1_:A_1_, **(b)** D_2_:A_1_ **)** and two ternary blends (**c, d** ), pump at 550nm, fluence 3.9×10^12^ photons/cm^2^.

**Supplementary Figures**

**a b**

**c**

**Supplementary Figure 4.** Excited-state dynamics in the infrared region. **a**. Transient dynamics of D1 only. **b.** Transient dynamics of D1:A1 blend. **c**. Transient dynamics of Ternary(H) blend. For these infrared transient tests, pump at 700 nm. Three main spectral features are detected in (**c**): the SE band in the spectral range 850-900 nm that is rapidly quenched by the formation of the interfacial charge transfer states (CT) PIA. The PIA peaked at 1140 nm assigned to charge carrier absorption, that grows in the first 300 fs and the PIA band peaking at 1500 nm assigned to singlet states, that shows a decay that matches both CT and charge carrier PIA rise. Such assignment consistent with previous reports^1, 2^.

**Supplementary Figures**

**a b c**

**Supplementary Figure 5.** Temperature dependent transient absorption of blends. **a.** Temperature dependent transient absorption spectra of D1: A1 (1:1) blends. **b.** Temperature dependent transient absorption spectra of Ternary(L) blends. **c.** Temperature dependent transient absorption spectra of Ternary(H) blends. Pump at 550nm, probe at 860nm, fluence 3.9×10^12^ photons/cm^2^. Calculate time constants accumulated in Supplementary Table 1.

**Supplementary Figures**

**a b**

**c**

**Supplementary Figure 6.** Fluence dependent transient absorption of blends. **a.** Intensity dependent transient absorption spectra of D1: A1 (1:1) blends. **b.** Intensity dependent transient absorption spectra of Ternary(L) blends. **c.** Intensity dependent transient absorption spectra of Ternary(H) blends. Pump at 550nm, probe at 860nm.

**Supplementary Figures**

**a b**

**Supplementary Figure 7.** Charge carrier and excited states kinetics of Ternary(H) and D1:A1 blends. **a**. Pump at 700 nm and probed at 1140 nm. Compared to D1:A1, there is faster charge carrier rising signal after 100 fs for the Ternary(H) blend. After global fitting, the rising time of Ternary (H) and D1:A1 is 67 fs and 134 fs, respectively. **b**. Pump at 700 nm and probed at 860 nm. For the excited species (charge transfer states), after 150 fs, the excited species generated faster and more efficient for the Ternary (H) blend. Both charge carrier and excited states kinetics results indicates that the ultrafast 100 fs process contributes the efficient charge generation in the Ternary (H) blend.

**Supplementary Figures**

**a**

**b**

**c**

**Supplementary Figure 8.** Electric field dependent transient luminescence and absorption for devices. **a.** Photoluminescence spectra under negative bias of Binary (D1:A1) and Ternary (H) devices. **b.** Transient absorption spectra under negative bias of Binary (D1:A1) and Ternary (H) devices, pump at 550nm and probe at 860nm. c. Transient absorption spectra under negative bias of Binary (D1:A1) devices, pump at 700nm and probe at 860nm. If there is some bound species, will be easily dissociate by applied bias for PL quenching test^3^. Even binary device demonstrates some PL quenching feature, while there is negligible quenching signature for Ternary(H) device, indicating excitons (bound species) in both devices cannot be influenced by the field. After negative bias(-3V) applied, there is significant PIA amplitude decreasing (~100fs) in the Ternary(H) device. Considering the PL quenching and field results, the difference in the initial kinetics under applied bias thus comes from photoinduced charged species.

**Supplementary Figures**

**a**

**b**

**
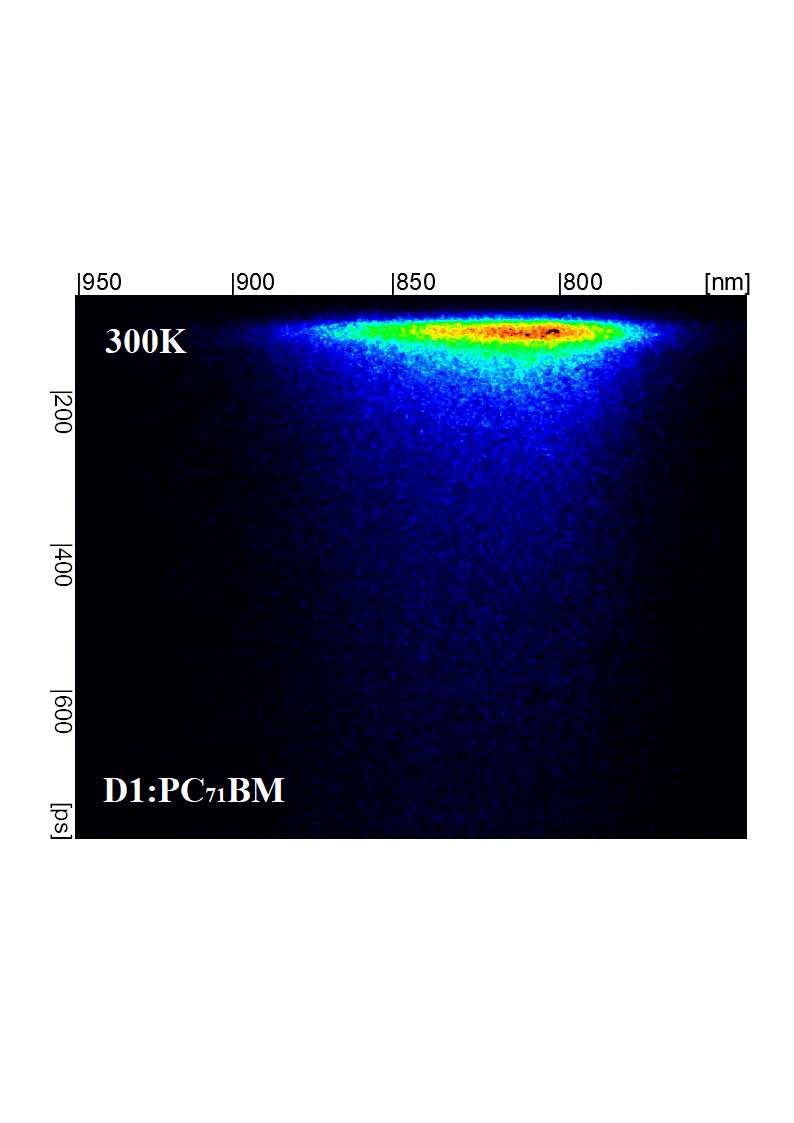

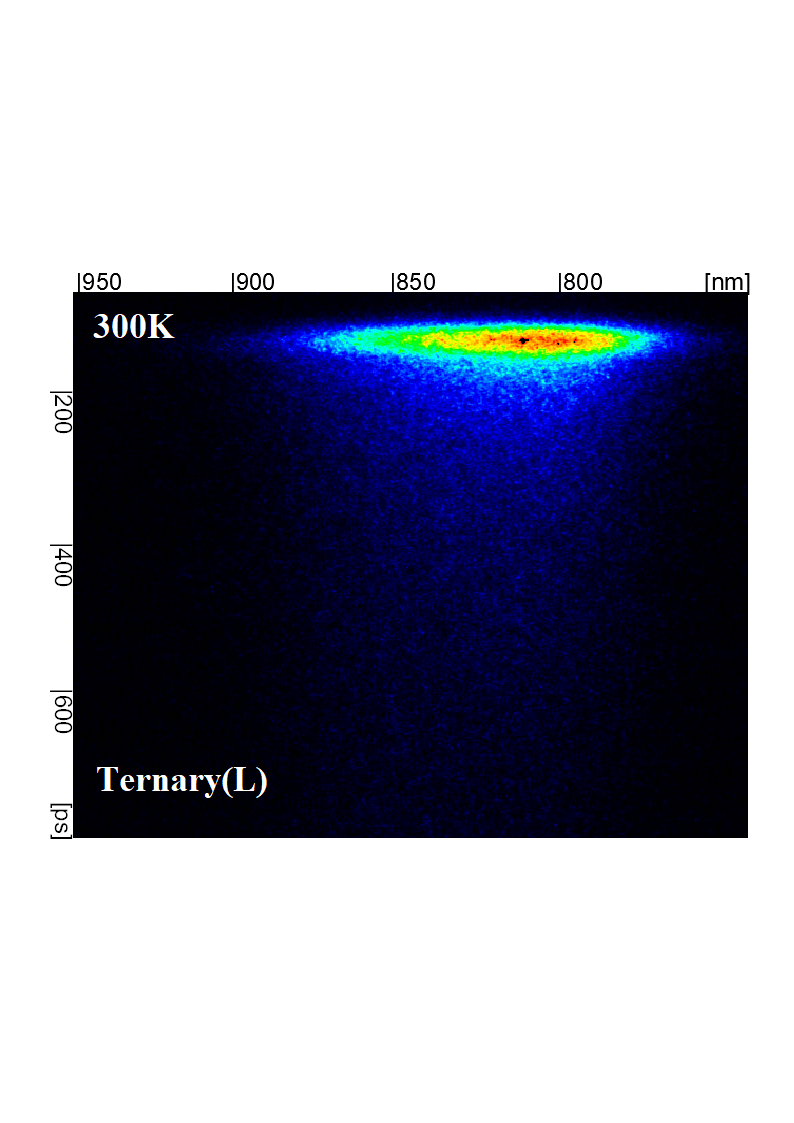

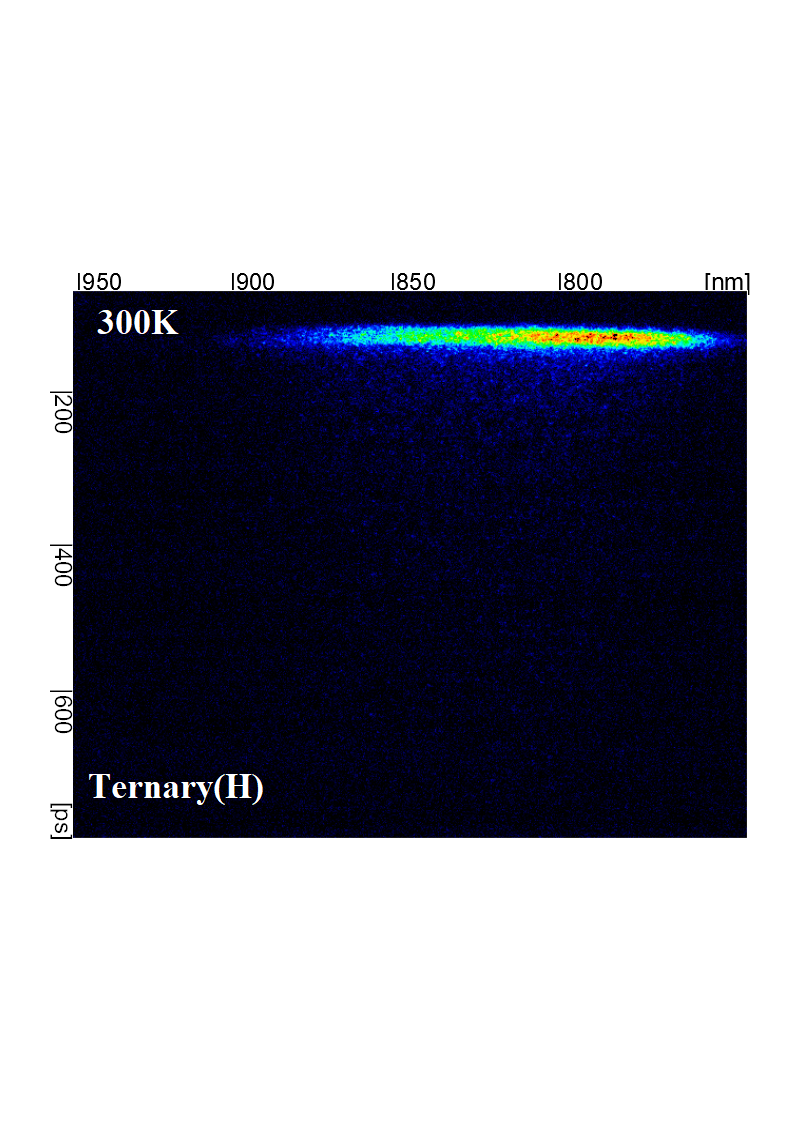
**

**c**

**
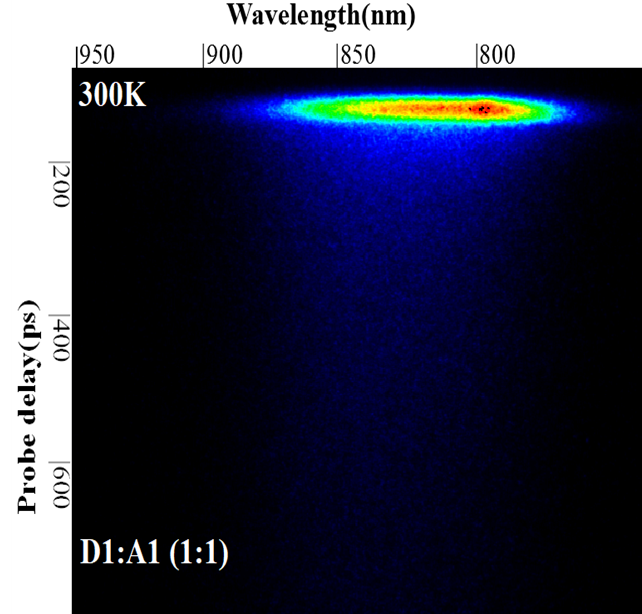

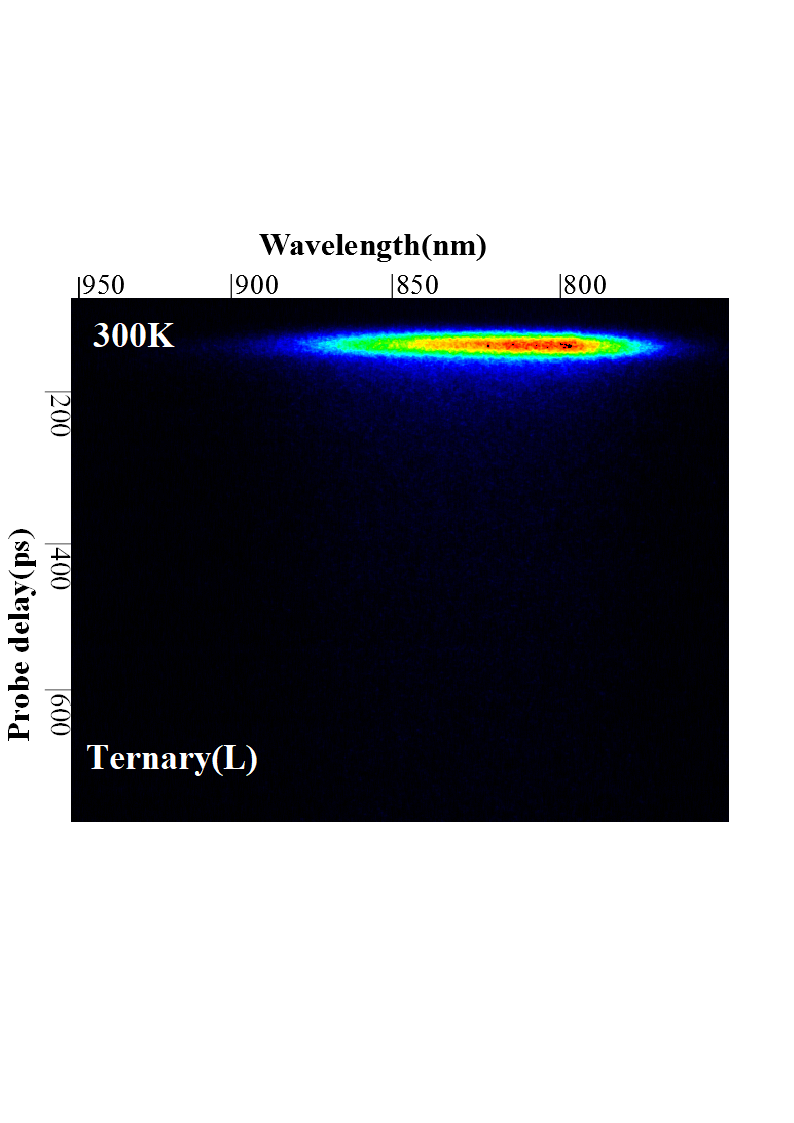

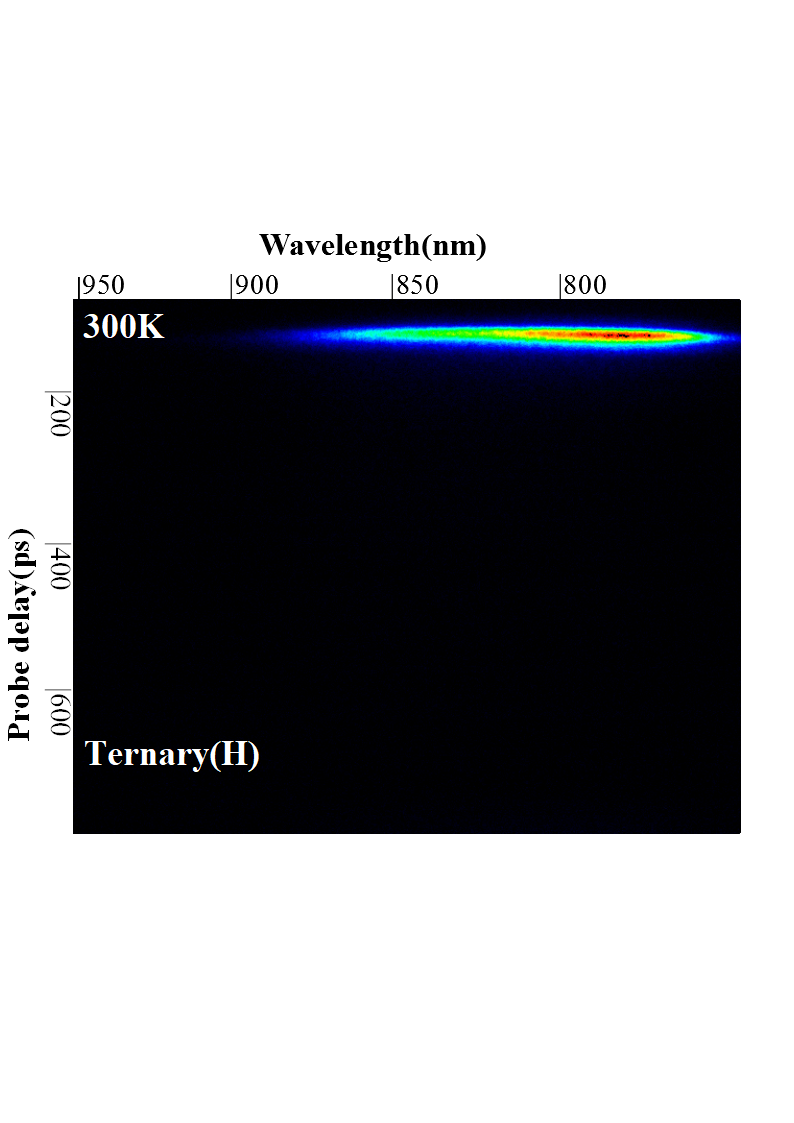
**

**Supplementary Figure 9.** Excited-state dynamics of pristine material and blends. **a**. Transient absorption kinetics of pristine D1 and Ternary (H) blends, pump at 550nm, probe at 860nm. For D1 only the initially-formed positive ESA signal is quickly replaced by SE, reflecting spectral migration-fast relaxation from the directly excited high to the lowest exciton states. After 0.5 picoseconds (ps), the kinetic curve represents the deactivation of the lowest-level excited state of D1. **b.** Time-resolved photoluminescence of three blends, pump at 445nm, probe at 740nm-950nm. **c.** Time-resolved photoluminescence of three blends, pump at 700nm, probe at 740nm-950nm.

**Supplementary Figures**

**a b**

**c**
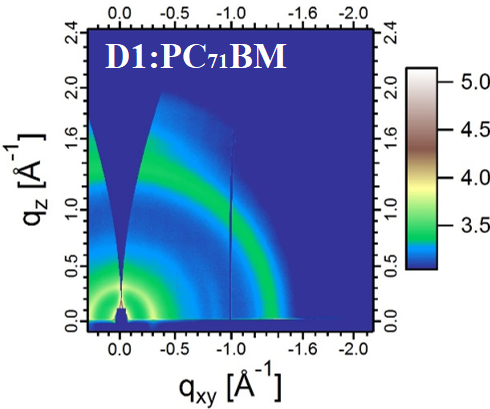

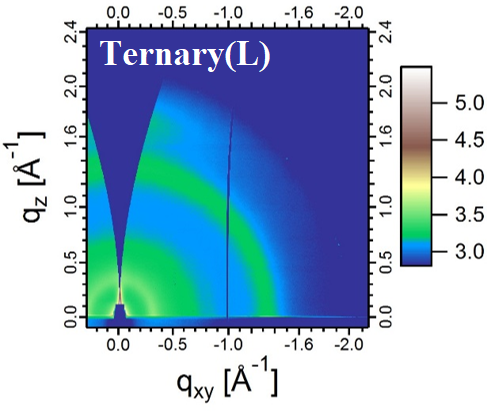


**
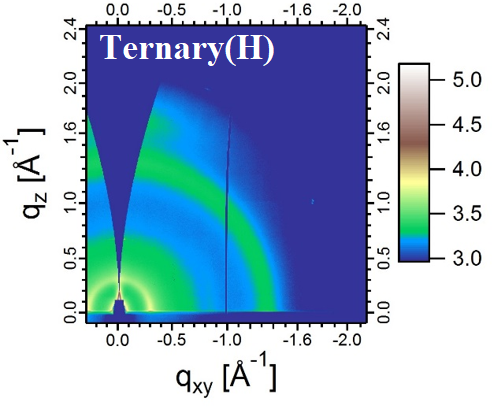
**

**Supplementary Figure 10.** Structural characterization and GIWAX spectra of blends. GIWAX spectra for **a.** D1:A1 (1:1) blends. **b.** Ternary(L) blends. **c.** Ternary(H) blends. It obvious PC_71_BM domain size (the d-spacing and CL value) is comparable in three different blends (Supplementary Table 2 and Supplementary Table 3).

**Supplementary Figures**

**a b**

**Supplementary Figure 11.** Interface area calculation for materials in devices. **a.** EQE spectra for three photovoltaic devices at room temperature. The CT absorption band is fitted with a Gaussian (FTPS) as described in the text. The results demonstrate the integrated signal of the CT band in the EQE spectrum, divided by the total integrated EQE(1.2eV~3.5eV)^4^ is comparable for three devices ((D1:A1):Ternary(L):Ternary(H) =2.2:2.1:2.3). **b.** Reduced CT EL spectrum for three photovoltaic devices. From the fitting results (Supplementary Note 3 and Supplementary Table 4), CT emission energy can be calculated using the same *E*_ct_ and λ values, which consistent with the experimental EL spectrum.

**Supplementary Figures**

**a b**

**c d**

**Supplementary Figure 12.** *J-V* curves for hole-only devices. **a.** *J-V* curves of hole-only devices for D1:PC_71_BM(1:1). **b.** *J-V* curves of hole-only devices for D1:D2:PC_71_BM (9:1:10), D2 with low molecular weight. **c.** *J-V* curves of hole-only devices for D1:D2:PC_71_BM (9:1:10). D2 with high molecular weight. d. *J-V* curves of hole-only devices for D1:D2:PC_71_BM (7:3:10). D2 with high molecular weight. The symbols are the raw data and the solid lines are fitting curves (Supplementary Note 4).

**Supplementary Figures**

**a b**

**c d**

**Supplementary Figure 13.** Disorder of hole-only devices**. a.** Temperature-dependent mobility values of hole-only devices for D1:PC_71_BM(1:1). **b.** Temperature-dependent mobility values of hole-only devices for D1:D2:PC_71_BM (9:1:10), D2 with low molecular weight. **c.** Temperature-dependent mobility values of hole-only devices for D1:D2:PC_71_BM (9:1:10). D2 with high molecular weight. **d.** Temperature-dependent mobility values of hole-only devices for D1:D2:PC_71_BM (7:3:10). D2 with high molecular weight. The symbols are the raw data and the solid lines are fitting curves, giving a disorder energy (Supplementary Note 5). Comparable disorder value obtained in these different devices.**Supplementary Figures**

**a b**

**c d**

**Supplementary Figure 14.** Resonance Raman spectra of pristine polymer and blends. **a.** Raman spectra in the low frequency region. **b.** Raman spectra in the high frequency region. **c.** Raman spectra for pristine PTB7-th.**d.** Raman spectra for Ternary(H) blend. The vibration mode demonstrates an almost temperature independence above 100K in the Ternary(H) blend. Films were prepared on silicon/native oxide substrates.

**Supplementary Figures**


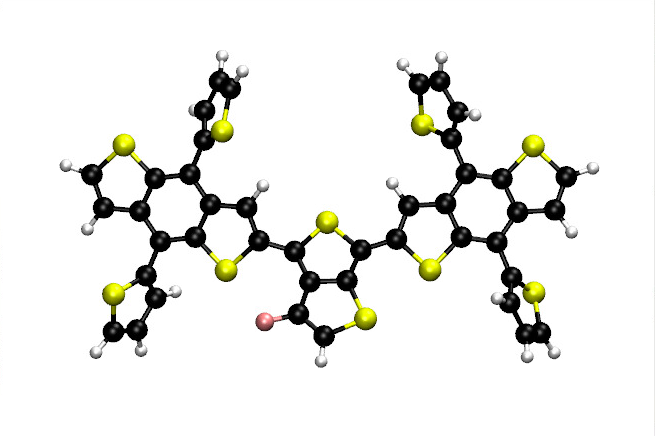

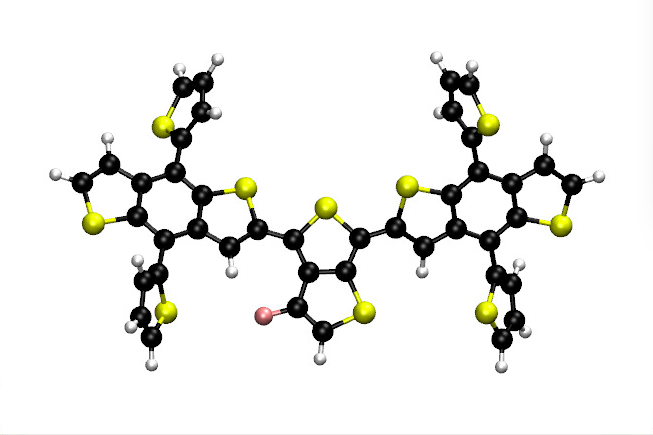


TT CC


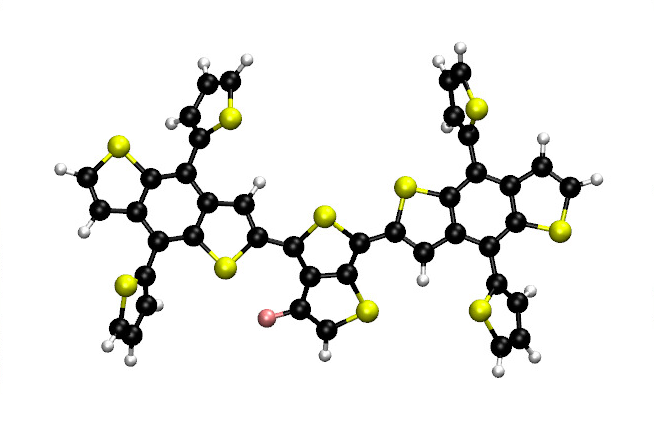

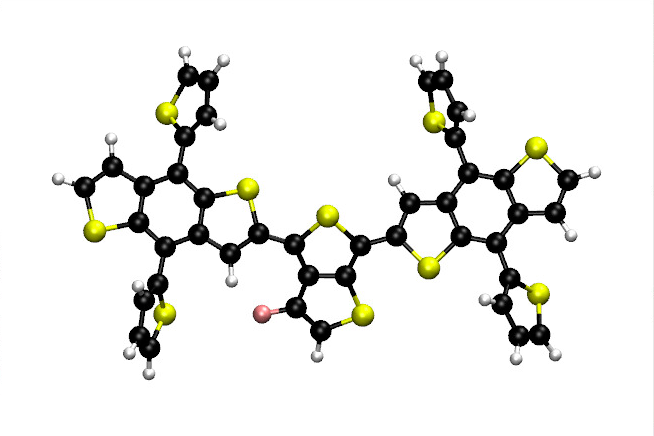


TC CT

**Supplementary Figure 15-1**. Model systems built to study the trans and cis conformation between the small and large units.

The relative energies obtained (Supplementary Table 5) show that the four conformers are relatively close to each other with a slight preference for the CT conformation.

**Supplementary Figures**

*Raman spectra*

For the four model systems presented in Supplementary Figure 15-1, we calculated the Raman spectra (Supplementary Figure 15-2). The four systems present an intense peak around 360-370 nm that we can attribute after visualization of the vibrations to a breathing mode of the large unit of PTB7-th.


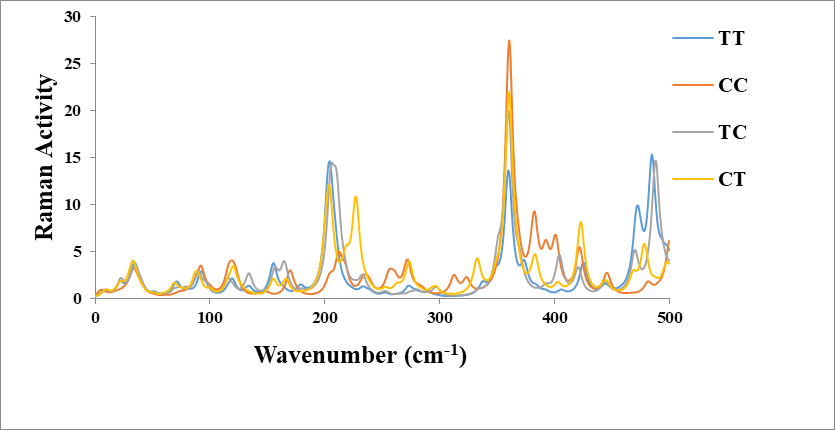


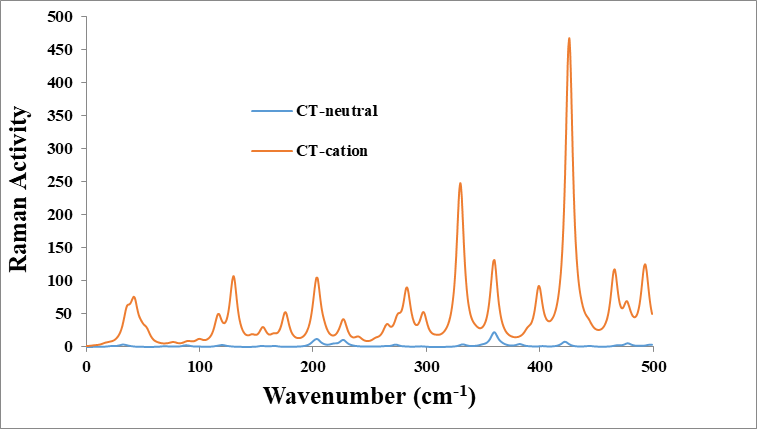


**Supplementary Figure 15-2.** Top: Raman spectra obtained at the B3LYP/6-31+G(d) level of theory for the four model systems presented. Bottom: Raman spectra of the neutral and radical cation of the CT (most stable conformer).

**Supplementary Figures**


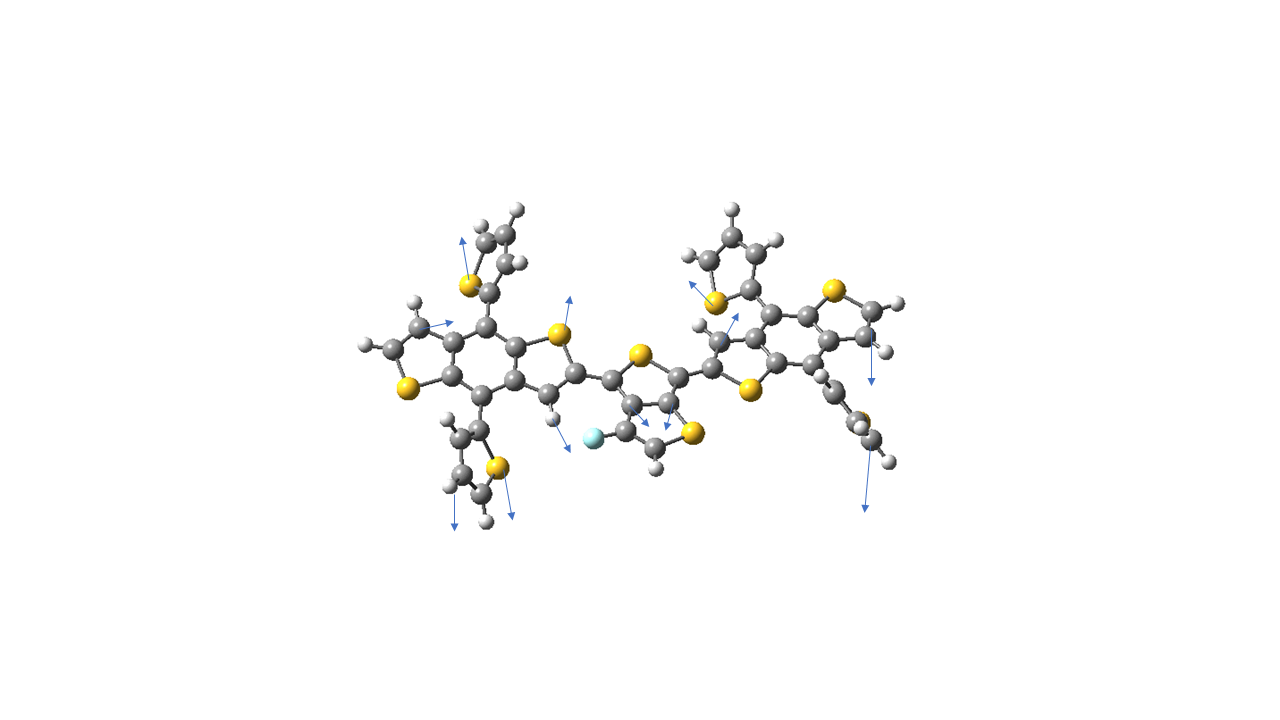

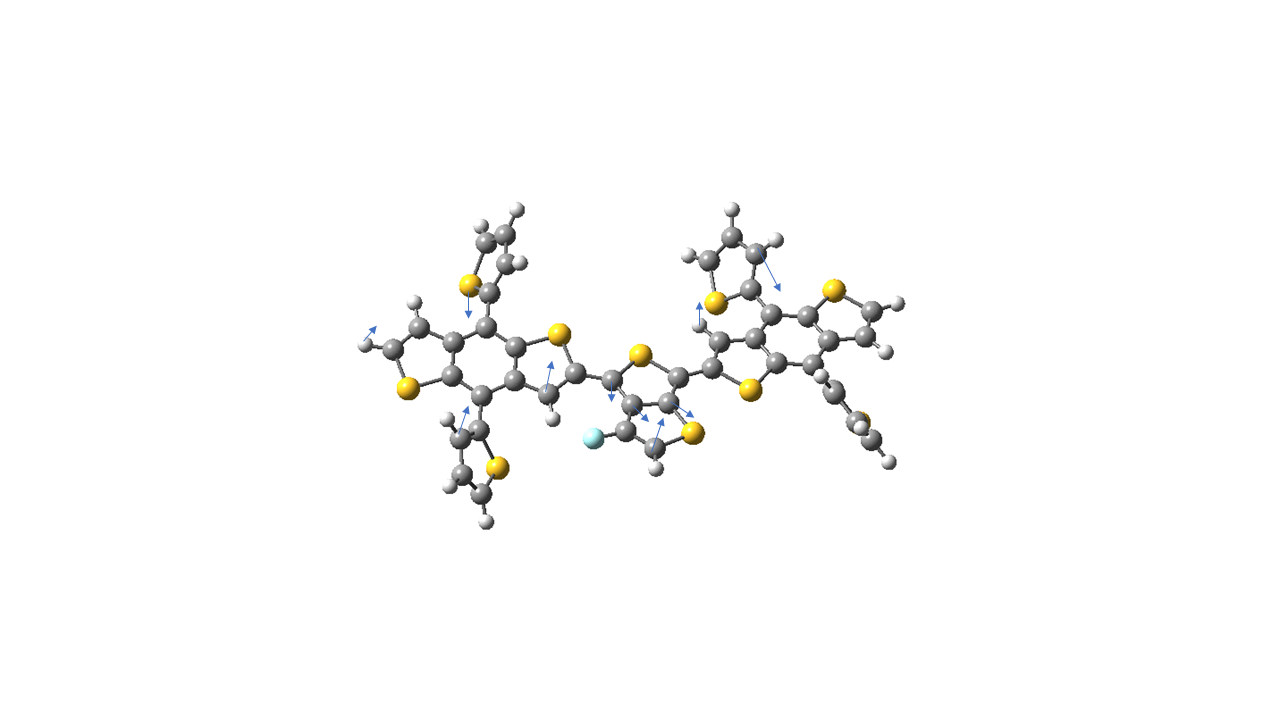


Mode 30 Mode 33


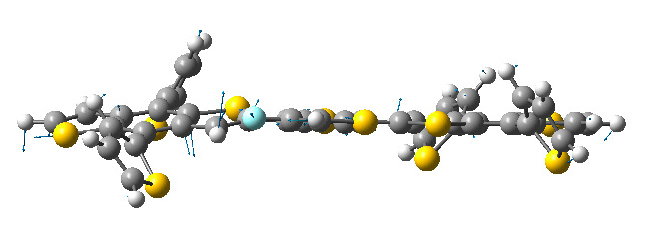

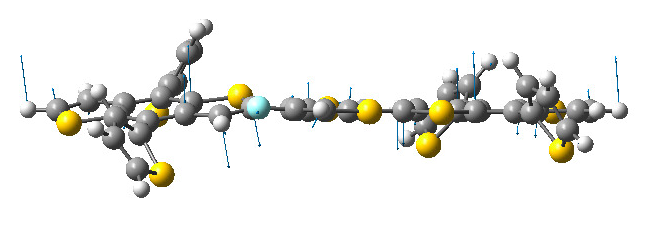


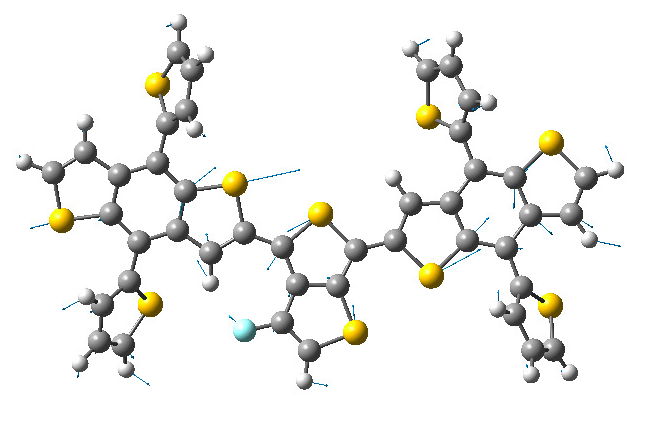
Mode 44 Mode 45


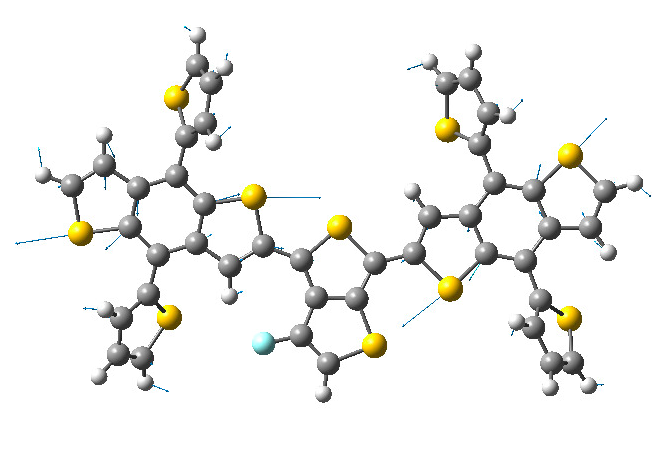


Mode 46 Mode 47


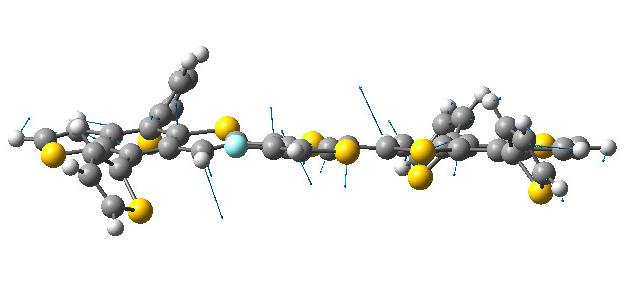

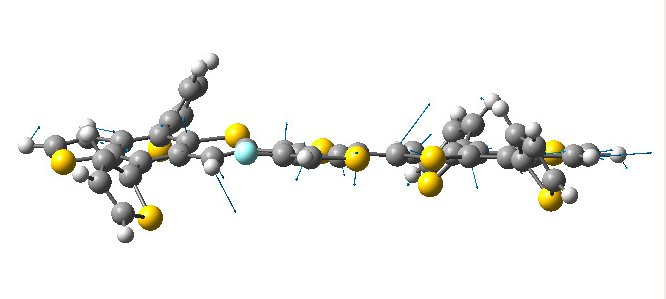


Mode 48 Mode 49

**Supplementary Figure 15-3.** Relative modes for the radical cation of the CT conformer. We observe a significative enhancement of the Raman activity form the neutral to the positively charge CT conformer (Supplementary Table 6), the mode 46 corresponding to the in plane breathing mode of the large unit of PTB7-th with a Raman activity passing from 28.8 to 168.7. Moreover, the mode 30 and 33 corresponding to the out-of-plane vibration of the central unit of PTB7-th with a Raman activity passing from 12.1 to 130.2 and from 10.5 to 50.3, respectively.

**Supplementary** **Figures**

**a b**

**c d**

**Supplementary** **Figure 16.** Temperature dependent time-resolved photoluminescence. Integrating the first 20ps of emission detected by a photon counting method, under room temperature (**a**) and 5K (**b**). **c.** PL dynamics of Ternary (H) with different times. **d.** PL decay of Ternary (H) blend, probed in the range 765-795nm. All measurements under 700nm pump and 0.1W fluence. In the Ternary(H) blend, due to rapid charge separation, D1 exciton having insufficient time to relax, results efficient CS states. Compared to initial exciton, these states demonstrate less Coulombic binding, results blue shift emission. Specially, there is no energy relaxation feature during its short lifetimes, rule out the interface orientation^5^ (between the two polymers D1 and D2) and aggregation contribution.**Supplementary** **Figures**

**a**


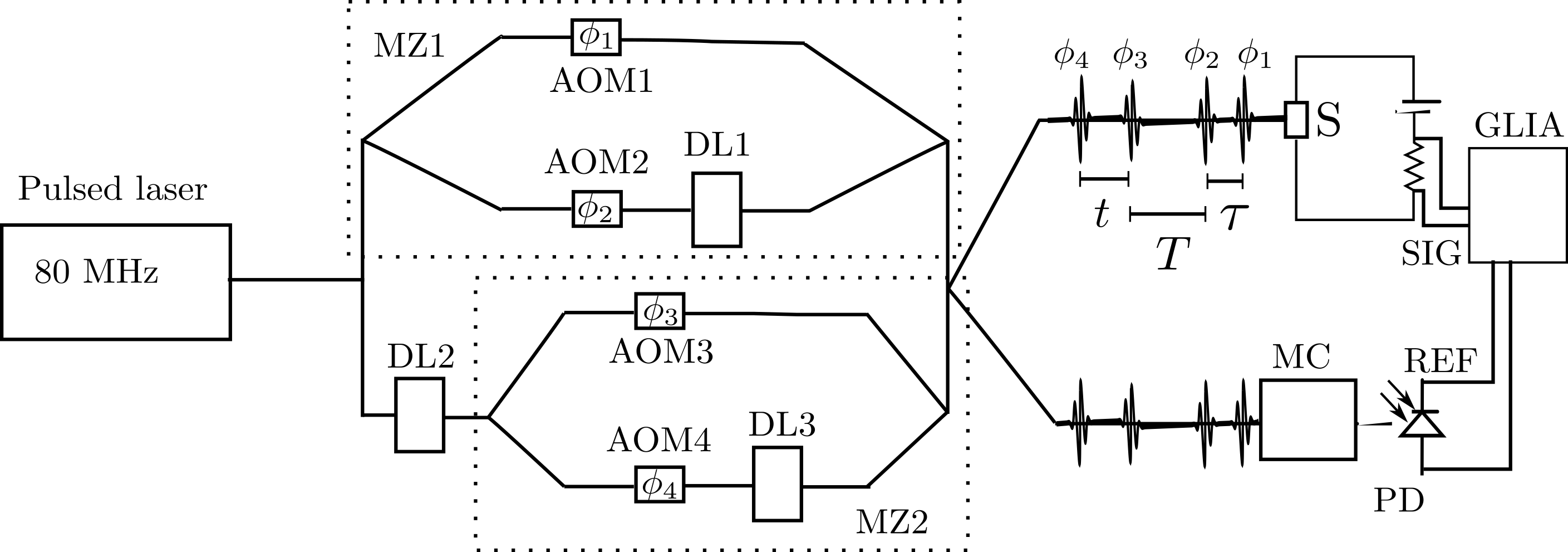


**b**


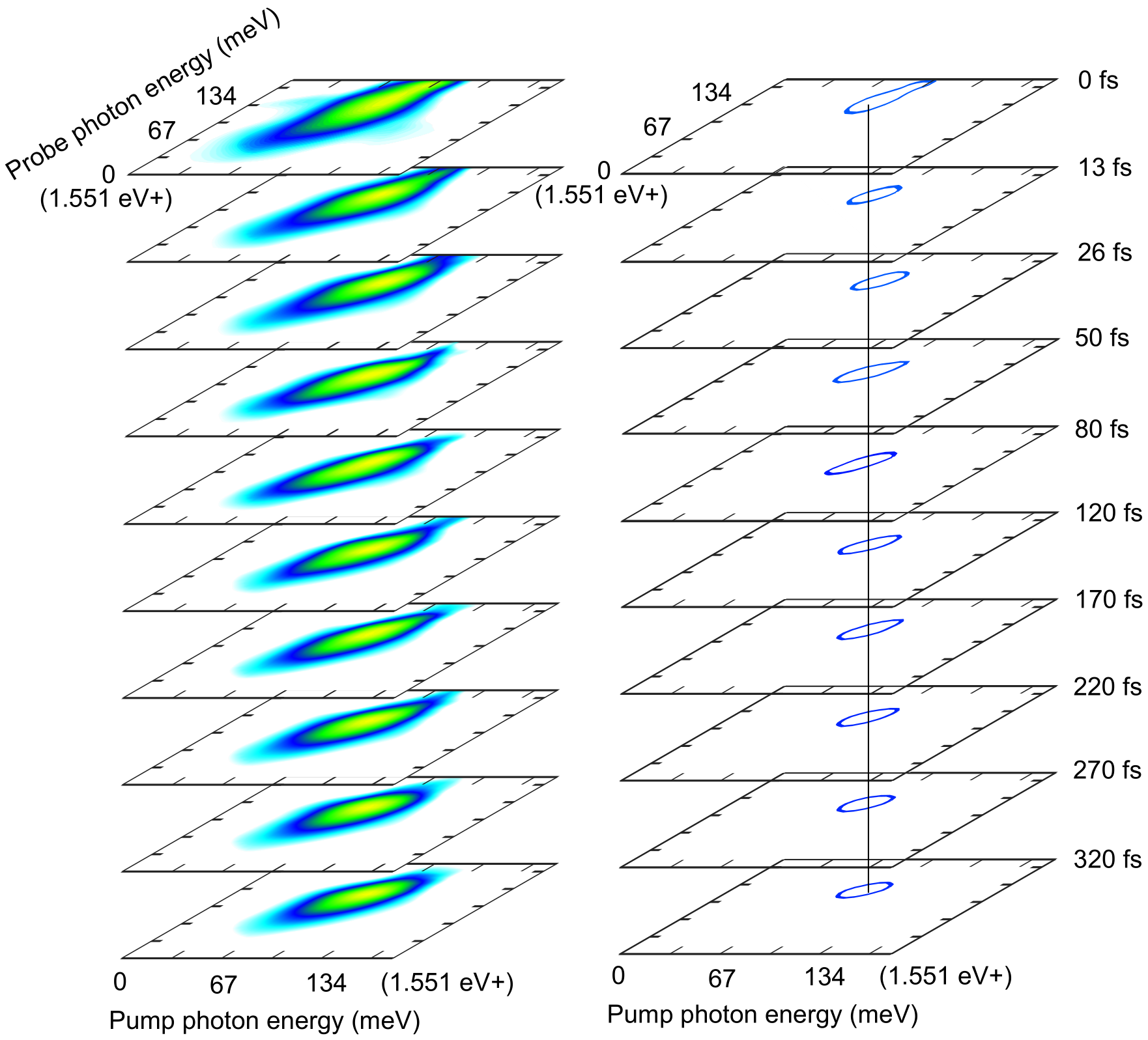


**c**


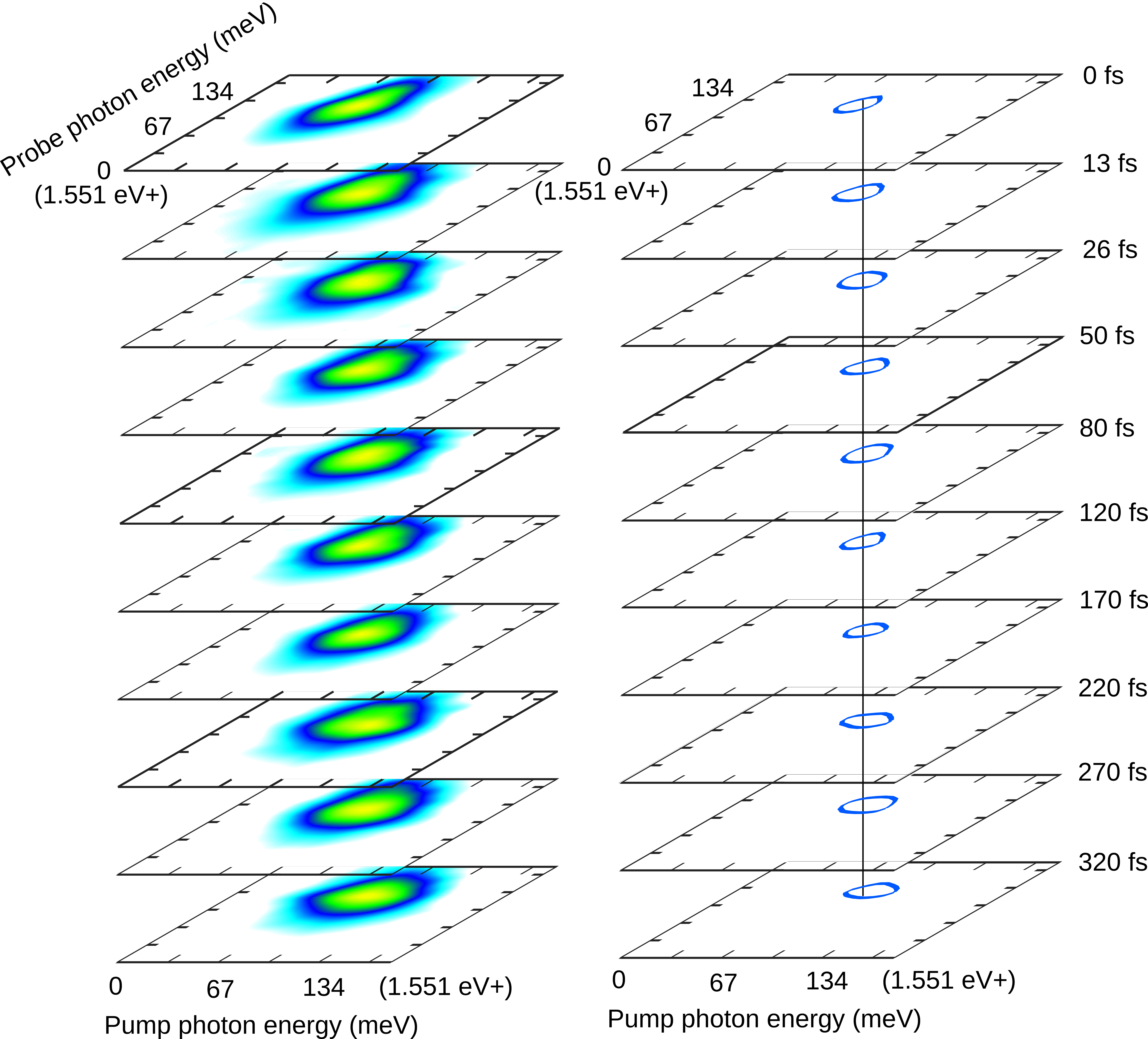


**Supplementary** **Figure 17**. **a.** Schematic of the setup used in the measurements of 2DPS. **b.** 2DPS for the Ternary (H) device and **c.** D1:PC_71_BM device. The left is the raw data and the right is the contour at 90% of the maximum amplitude. The Z axis is the population time.

**Supplementary** **Figures**


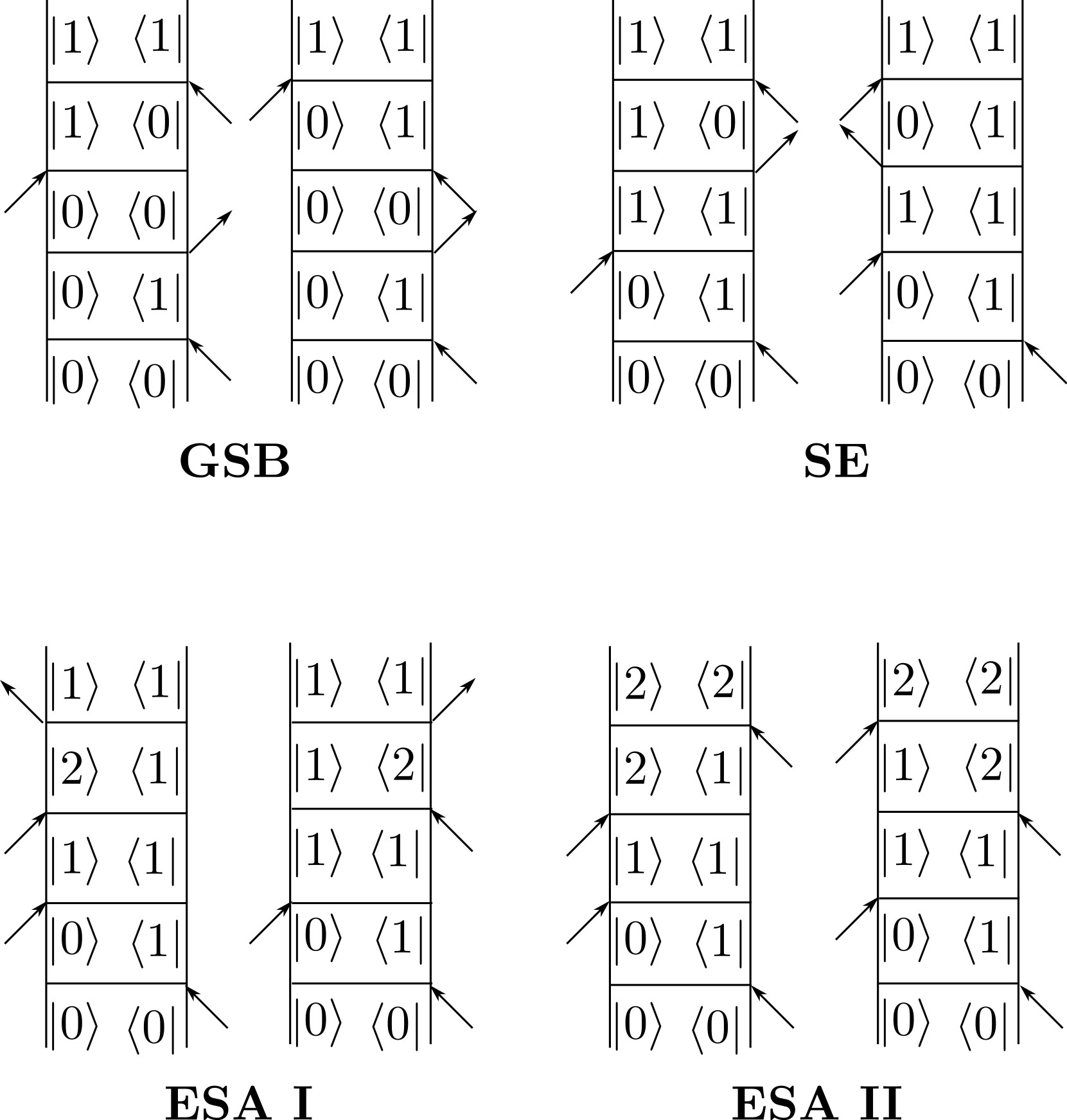


**Supplementary** **Figure 18.** The Feynman diagrams depicting the different light-matter interaction pathways. The spectra, in general, have contributions from three types of signals, the ground state bleach (GSB), stimulated emission (ESA) and excited state absorption (ESA). As opposed to the traditional type of 2D spectra where one detects coherent four-wave mixed signal, photocurrent 2D spectra have two types of ESA signals, which are often called as ESA I and ESA II. The Feynman diagrams depicting the different light-matter interaction pathways are given in Fig. 3g. The diagrams show the evolution of the density matrix of the system when perturbed by the light. The ESA I and ESA II differ in the final state that is reached after the four interaction. In ESA I, the system is finally in the first excited state |1>, while in ESA II it is in the high lying excited state |2>. Each of the signals have a sign associated to it, which is given by (-1)*^n^*^-1^, where *n* is the number of interactions from the bra (or the ket) side of the density matrix^6^. According to the diagrams, the GSB, SE and ESA I have the positive sign while the ESA II has the negative sign. In most of the solar cells, the excitation of high lying states does not yield higher photocurrent yield compared to the excitation at the band-edge because the excess energy is dissipated as heat due rapid thermal relaxation. Consequently, the ESA I and ESA II signals cancel each other, such that the 2D photocurrent signal effectively comes from the GSB and SE pathways. Spectra from these two pathways have only absorptive lineshapes with only positive peaks that are similar to the ones we observe in our 2D photocurrent measurements.

**Supplementary** **Figures**

**a**

**b**

**
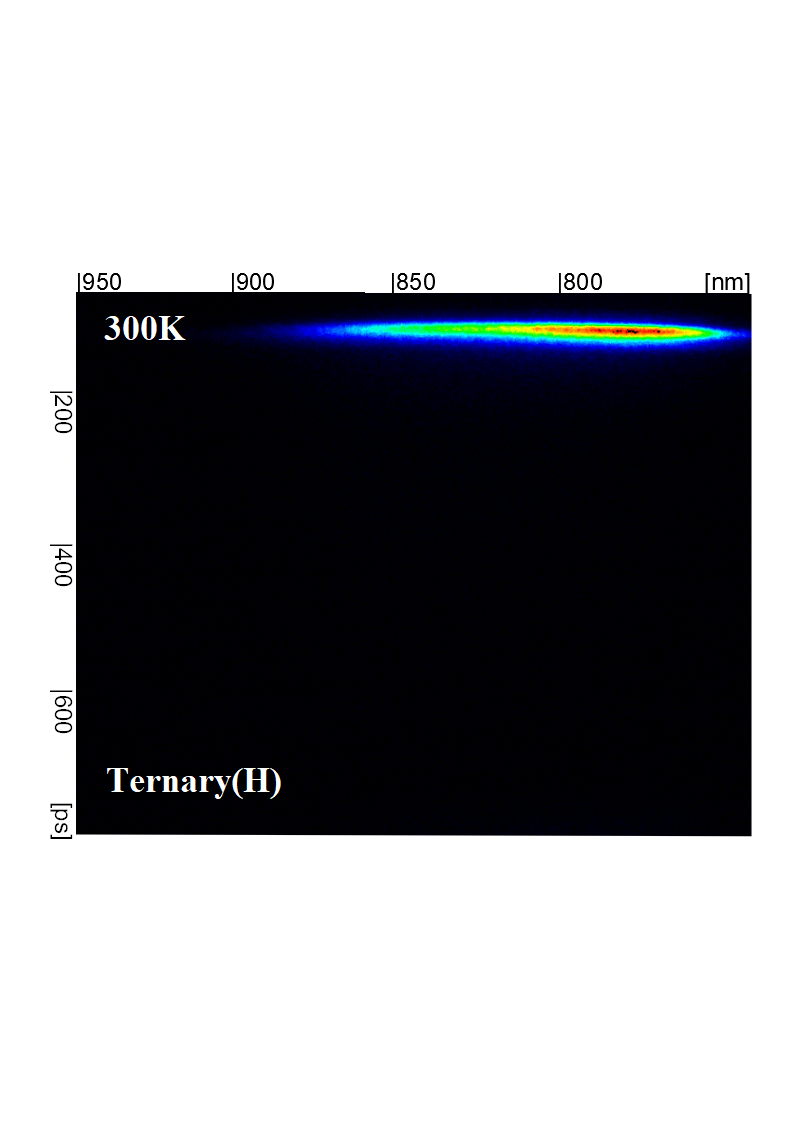

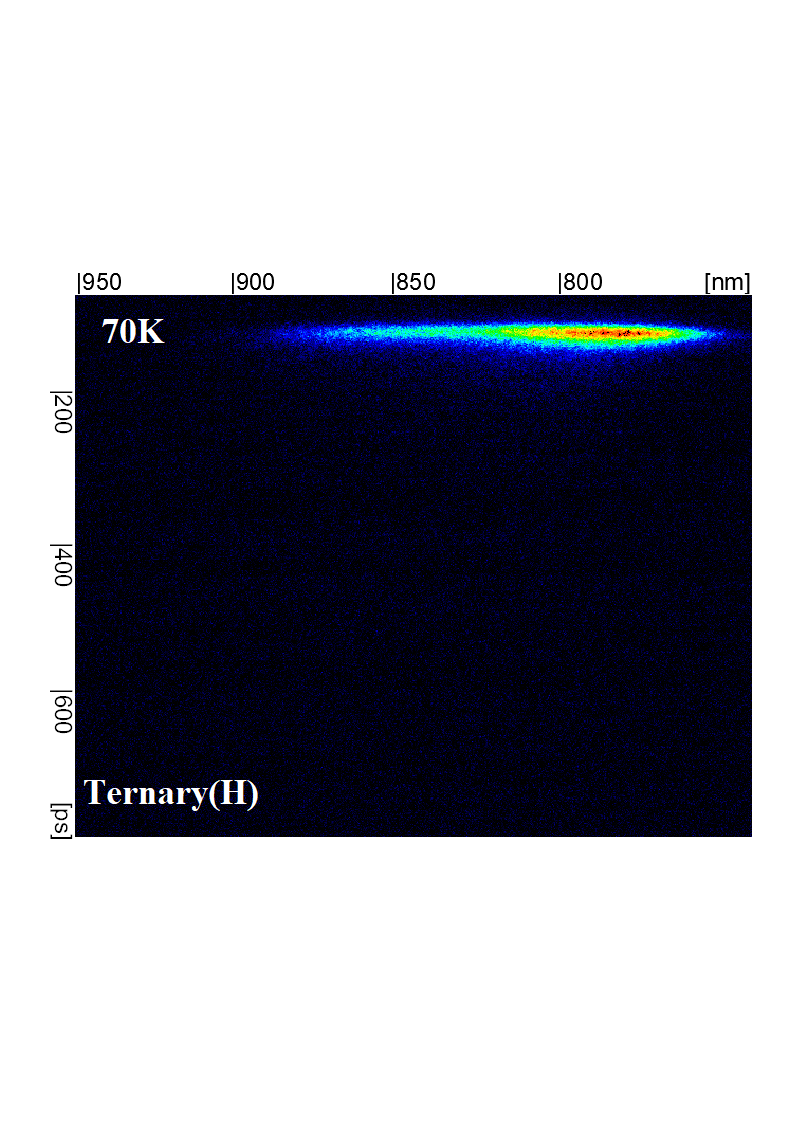
**

**
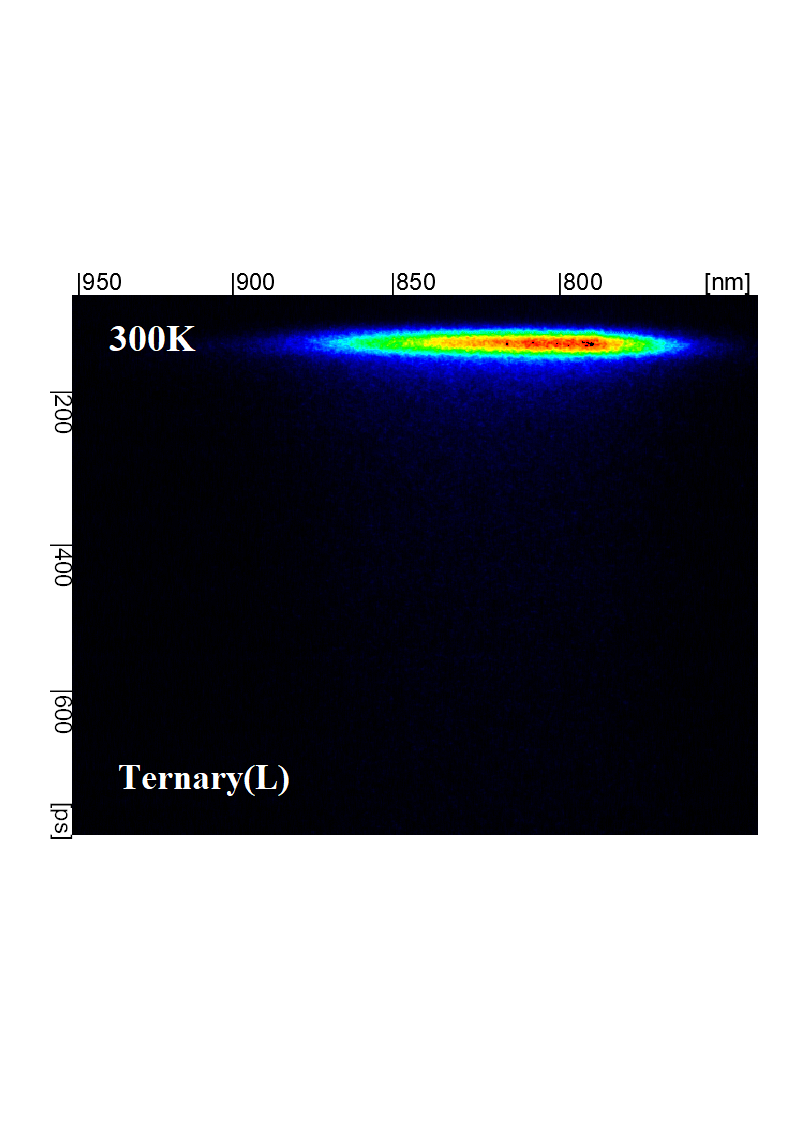

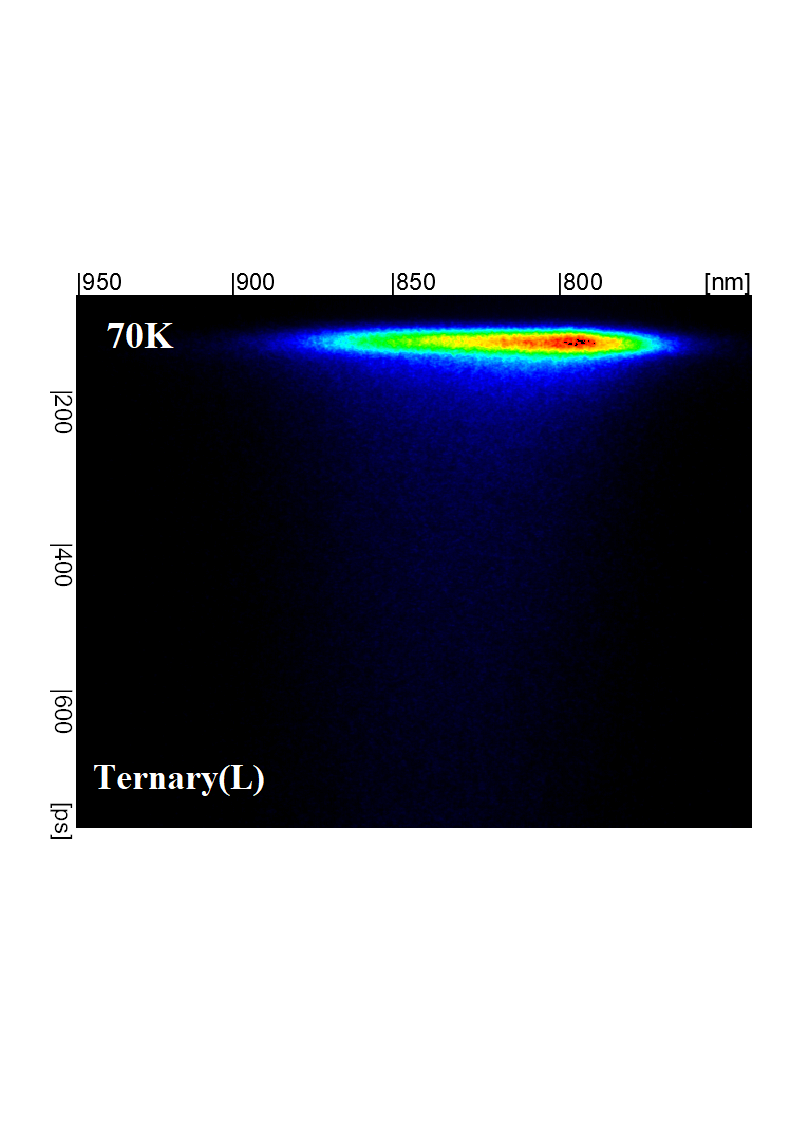
**

**
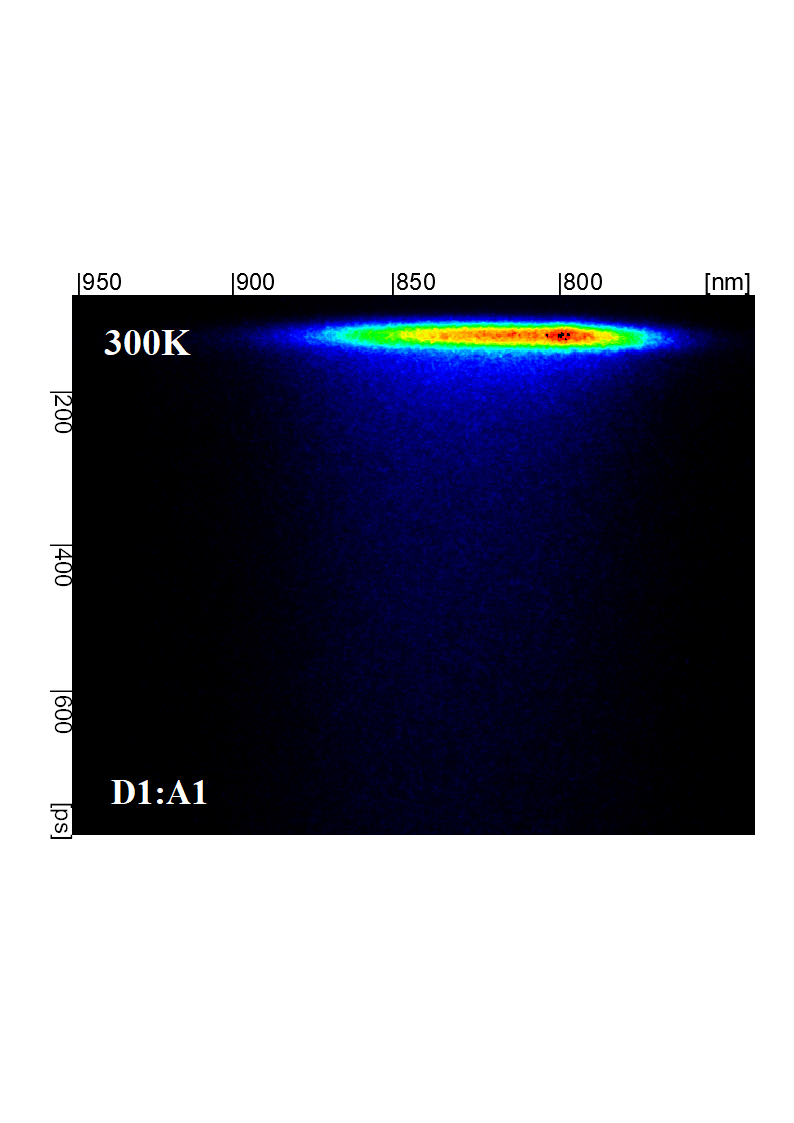

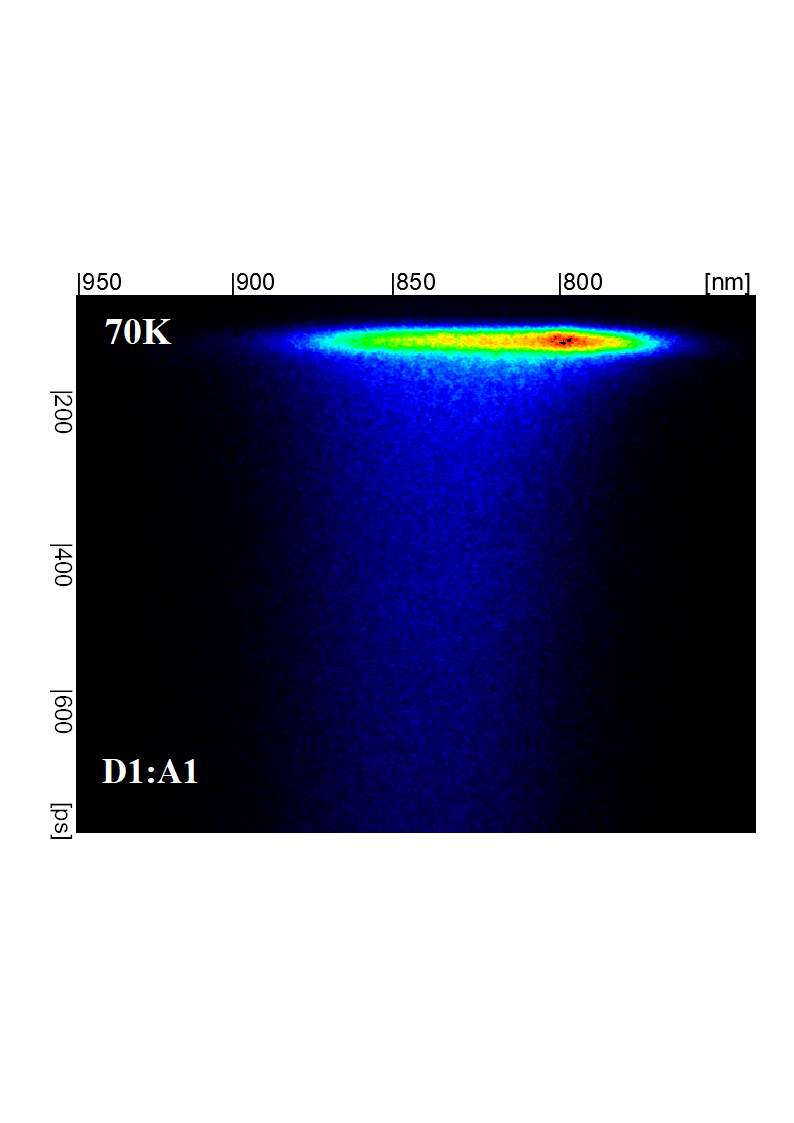
**

**c**

**
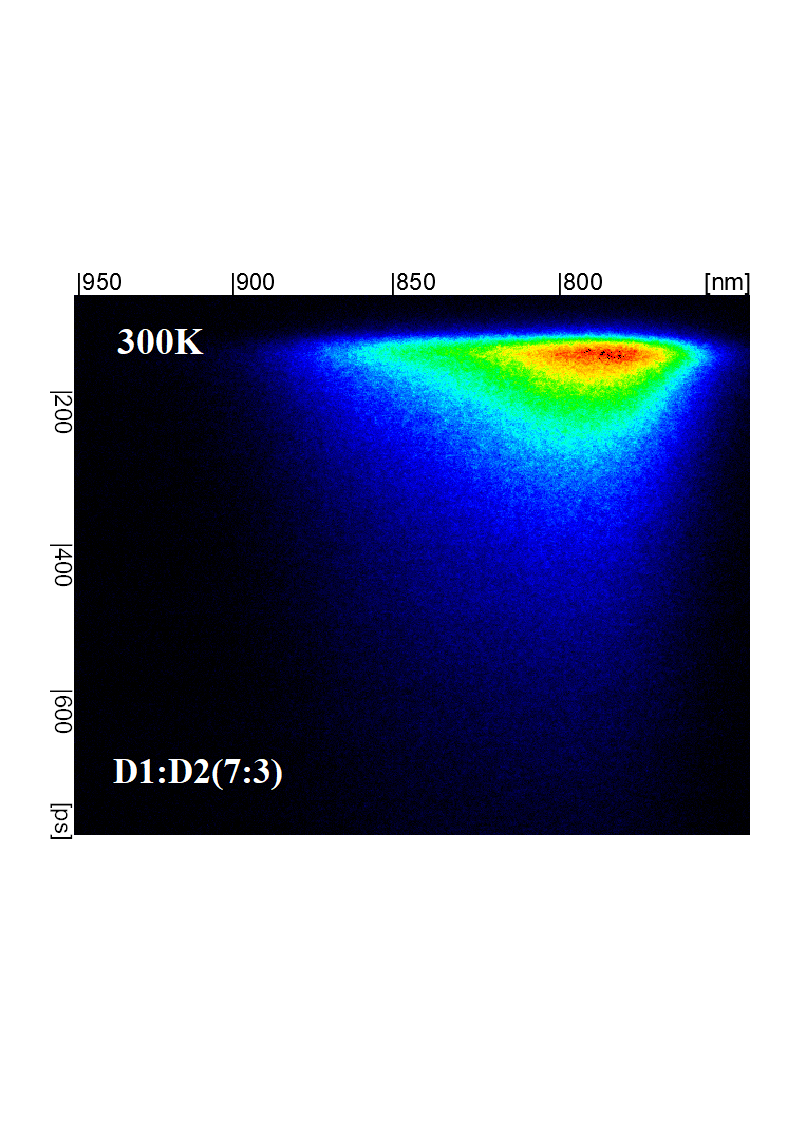

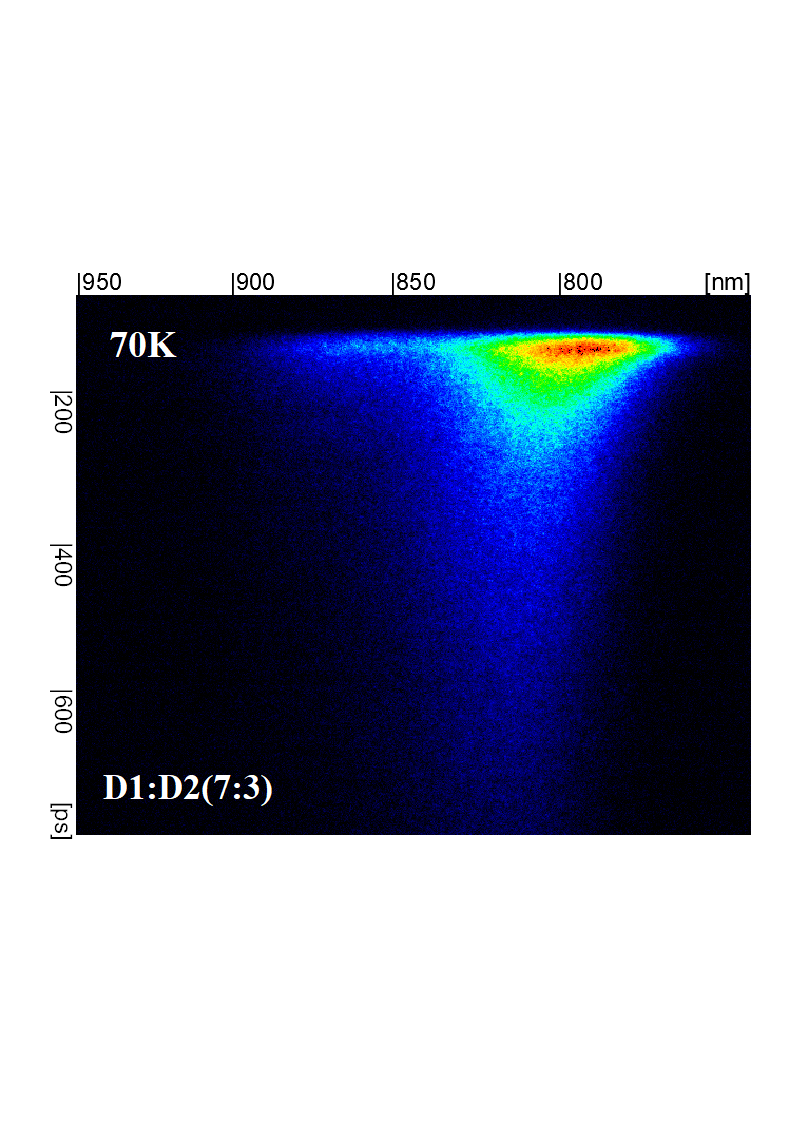
**

**d**

**
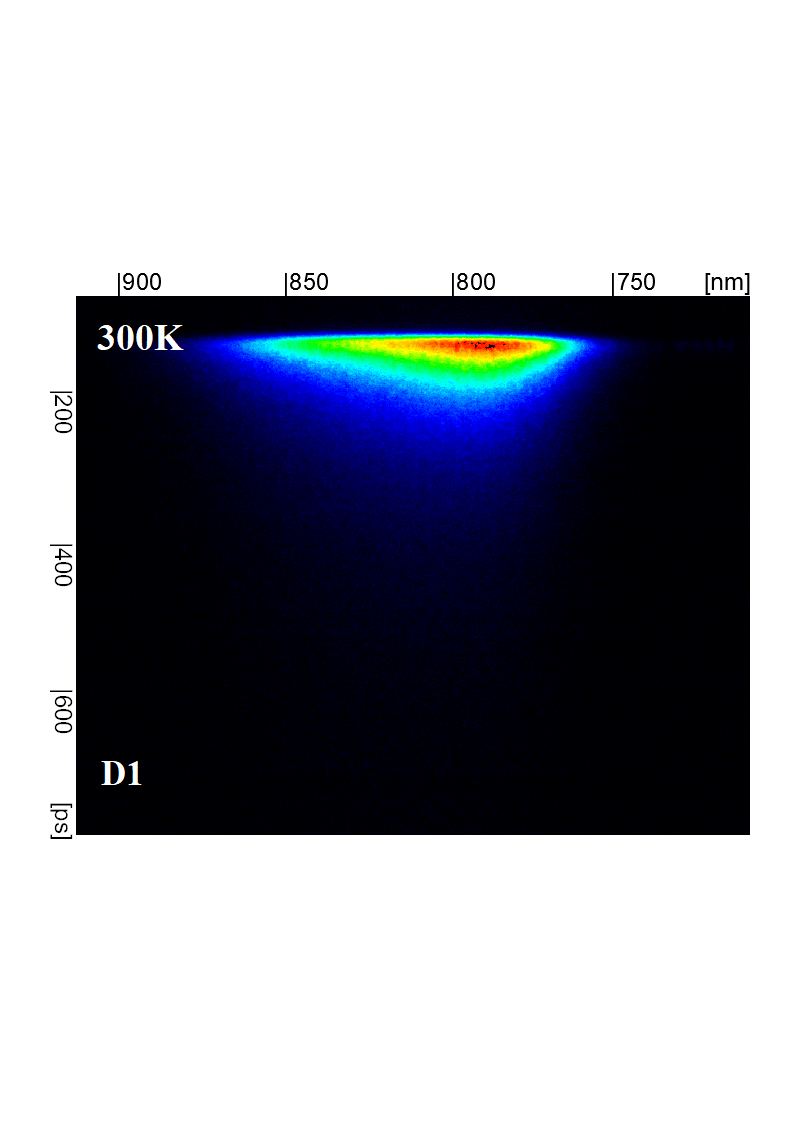

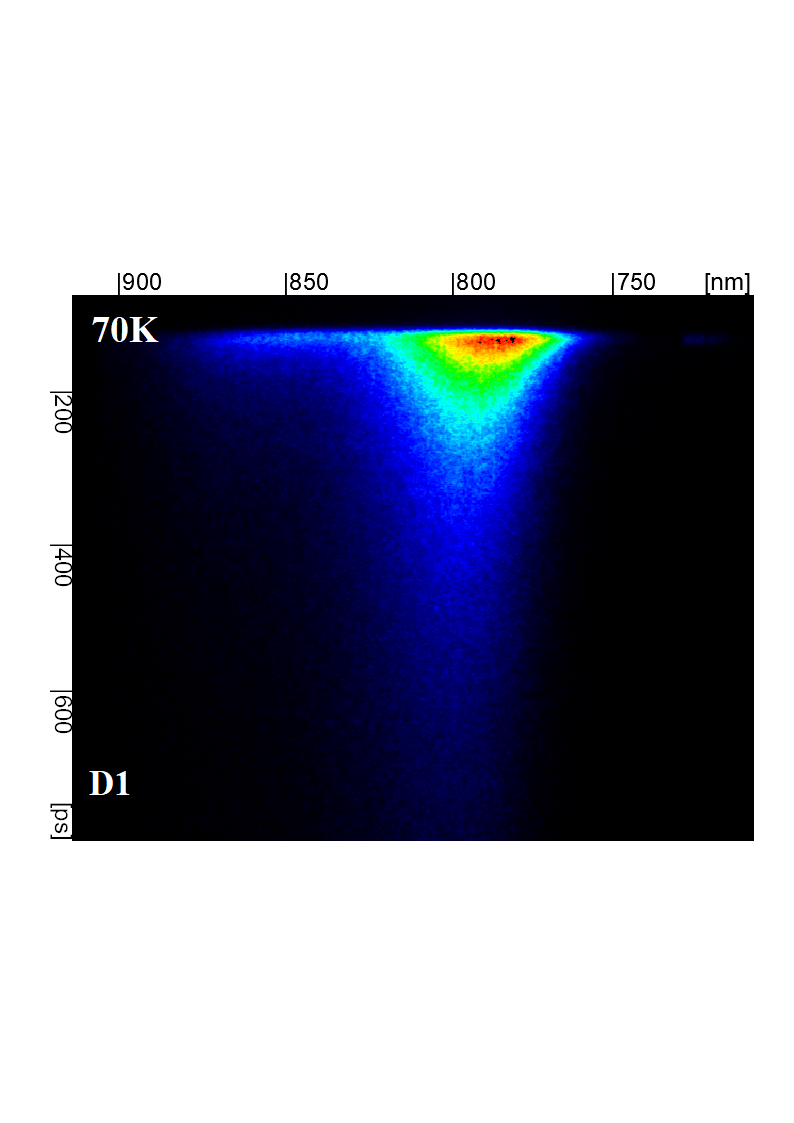
**

**Supplementary** **Figure 19.** Energy transfer process for fullerene-based blends. **a.** Transient absorption data of the Ternary (H) blend, Ternary (L) blend and D1:A1(1:1) blend. Pump at 550nm, fluence 3.9×10^12^ photons/cm^2^. It is known that the excitation energy transfer from the excited conjugated segments results in a dynamical Stokes shift, exhibiting a redshifted emission tail^7^. The Ternary (L) and Binary D1:A1 blend show a significant peak redshift at initial 200fs, indicating efficient energy transfer, while this is not observed in the Ternary (H) blend (Supplementary Note 6).

**Supplementary Figures**

**a b**

**Supplementary Figure 20.** Characterization of devices based on ITIC. **a.** J-V curves of device. **b.** V_oc_-T plots for the binary and ternary devices. Using the same molecular weight and donor ratio as used in A1 based blends, we fabricate the Binary and Ternary blend. Devices based on these blends performed well with PCE ≈6%. Unlike PC_71_BM blends, there is no significant difference after **D2** is added in a ternary blend.

**Supplementary Figures**

**a**

**b**

**c**

**Supplementary Figure 21.** Transient dynamics for ITIC blends and devices. **a.** Transient absorption data for ITIC based binary blend. **b.** Transient absorption data for ITIC based ternary blend. Both (a) and (b) pump at 550nm, fluence 3.9×10^12^ photons/cm^2^. **c.** Transient absorption data for ITIC based binary and ternary devices, pump at 700nm. Here, the signal around 940nm in transient absorption come from ITIC excited state^8^. At the initial timescale, significant redshift in both blends and no bias dependent feature in both devices, indicating there is no initial charge transfer process for both blends.

Supplementary Table 1| Time constants of different kinetic components obtained by fitting the kinetic curves with a multi-exponential function.

| T(K) | Rise1(fs) | Rise2(ps) | Decay2(ps) |  |
| --- | --- | --- | --- | --- |
| 290 | 30 | 5.5 | 1724 | D1: PC_71_BM  1:1 |
| 230 | 30 | 7.9 | 2531 |  |
| 170 | 30 | 9.4 | 4276 |  |
| 110 | 30 | 10.9 | 5842 |  |
| 77 | 30 | 11.2 | 9000 |  |

| T(K) | Decay1(fs) | Rise2(ps) | Decay2(ps) |  |
| --- | --- | --- | --- | --- |
| 290 | 50 | 4.2 | 1800 | Ternary(L) |
| 230 | 50 | 5.9 | 2899 |  |
| 170 | 50 | 8.6 | 5290 |  |
| 110 | 50 | 9.9 | 11048 |  |
| 77 | 50 | 12.8 | 21734 |  |

| T(K) | Decay1(fs) | Rise2(ps) | Decay2(ps) |  |
| --- | --- | --- | --- | --- |
| 290 | 50 | 2.0 | 1580 | Ternary(H) |
| 230 | 50 | 2.7 | 1818 |  |
| 170 | 50 | 3.3 | 2929 |  |
| 110 | 50 | 3.6 | 4506 |  |
| 77 | 50 | 3.7 | 6706 |  |

The ultrafast decay (Decay 1, observed in the Ternary blend), demonstrates a Temperature independent feature.

Supplementary Table 2| Fitting Parameters for GIWAX of Blends

|  | In plane(PCBM) | | | |
| --- | --- | --- | --- | --- |
|  | location(Å^-1^) | d-spacing(Å) | FWHM | CL(nm) |
| Ternary(L) | 1,3197 | 4,7610 | 0,2346 | 24,1022 |
| Ternary(H) | 1,3240 | 4,7456 | 0,2309 | 24,4905 |
| D1: PC_71_BM (1:1) | 1,3306 | 4,7220 | 0,2335 | 24,2157 |

Supplementary Table 3| Fitting Parameters for GIWAX of Blends

|  | In plane(100) | | | |
| --- | --- | --- | --- | --- |
|  | location(Å^-1^) | d-spacing(Å) | FWHM | CL(nm) |
| Ternary(L) | 0,3067 | 20,4837 | 0,0868 | 65,1227 |
| Ternary(H) | 0,2890 | 21,7381 | 0,0546 | 103,4080 |
| D1: PC_71_BM(1:1) | 0,2934 | 21,4107 | 0,0676 | 83,6024 |

Supplementary Table 4| FTPS fitting results

|  | f (eV^2^) | *E*_ct_ (eV) | λ (eV) | *E*_el_ (eV) |
| --- | --- | --- | --- | --- |
| D1:A1 | 3.0E-2 | 1.43 | 0.33 | 1.10 |
| Ternary(L) | 1.5E-2 | 1.38 | 0.33 | 1.05 |
| Ternary(H) | 2.0E-3 | 1.31 | 0.28 | 1.03 |

Supplementary Table 5: Relative energies for the conformers

| Conformers | Relative Energies (kcal/mol) |
| --- | --- |
| TT | 0.53 |
| CC | 0.10 |
| TC | 0.48 |
| CT | 0.00 |

Supplementary Table 6: Assignment of the modes for the neutral and cation of CT conformer

|  | **CT neutral** | | **CT cation** | |  |
| --- | --- | --- | --- | --- | --- |
| Mode # | Wavenumber (cm^-1^) | Raman Activity | Wavenumber  (cm^-1^) | Raman Activity | Assignment |
| 30  33  44 | 203.0  226.0  332.1 | 12.1  10.5  9.1 | 203.2  226.6  329.9 | 130.2  50.3  272.0 | out-of-plane vibration of the central unit  out-of-plane vibration of the central unit  out of plane of the large unit |
| 45 | 349.1 | 1.7 | 343.1 | 24.8 | out of plane of the large unit |
| 46 | 359.8 | 28.8 | 359.6 | 168.7 | in plane breathing mode of the large unit |
| 47 | 366.7 | 17.4 | 366 | 9.6 | in plane breathing mode of the large unit |
| 48 | 383.4 | 5.1 | 382 | 1.2 | out of plane of the large unit |
| 49 | 391.3 | 2.9 | 390 | 98.1 | out of plane of the large unit |

**Supplementary Note 1:** Compared to the Binary device (D1:PC_71_BM), there is an enhanced photocurrent (Jsc) and fill factor (FF) for ternary devices. For indication case, in the main and Supplementary Materials text, the optimized blends are **Ternary (H)** (PTB7-th:PCDTBT(H):PC_71_BM (7:3:10)，PCDTBT with high molecular weight) and **Ternary (L)** (PTB7-th:PCDTBT(L):PC_71_BM (9:1:10)，PCDTBT with low molecular weight).

**Supplementary Note 2:** For D1and D1: D2 (7: 3) films, three main spectra features are detected. The stimulated emission (SE) band in the spectral range 830-880nm. The excited state absorption (ESA) and the photo-induced absorption (PIA) band in the spectral range 880-950nm.

These results are consistent with energy transfer (FRET) theory, an efficient energy transfer process cause an increased population of excited states in the energy acceptor^9^. This conclusion can also be confirmed by time-resolved photoluminescence (TRPL), as illustrated in b, the PTB7-th excited state emission increased in the blend. The Förster resonance energy transfer (FRET) is established in the D1-D2 system with enhanced excited state population in D1^10, 11^.

It should be noted here that the increased population of D1 excited states are available for the ultrafast transfer process to PC_71_BM; this enables us to explore the primary charge delocalization and transfer process.

**Supplementary Note 3:** The CT region of the EQE spectra obtained by using Fourier transform photocurrent spectrum (FTPS)^12^, and fits the CT band using Equation

$EQE(E)\propto\frac{f_{\sigma}}{E\sqrt{4\pi\lambda kT}}exp\left( \frac{{-\left( E_{ct}+\lambda-E \right)}^{2}}{4\lambda kT} \right)$ (2)

Where *k* is the Boltzmann’s constant, *T* is the absolute temperature, $E_{ct}$ is the energy of CT state, $f_{\sigma}$ is proportional to the square of the electronic coupling matrix element, and *λ* is related to the width of the CT absorbance band, with contribution from internal/environmental reorganization and/or energetic disorder.

**Supplementary Note 4:** The mobility was determined by fitting the Single-carrier *SCLC*, which described by the equation (3)

$$J=\frac{9}{8}\varepsilon_{0}\varepsilon_{r}\mu\frac{V^{2}}{d^{3}}$$

where *J* is the current, *μ* is the charge mobility at zero field, ε*_0_* is the free-space permittivity, ε*_r_* is the relative permittivity of the material, *d* is the thickness of the active layer, and *V* is the effective voltage *V*–*V_bi_*.

**Supplementary Note 5:** Using the mobility results fitting the voltage dependence, data is collected that can be used to continue using Equation (4)^13^

$\mu\left( T \right)=\mu_{0}exp\left[ -\left( C\frac{\sigma}{kT} \right)^{2} \right]$ (4)

Where *k* is the Boltzmann’s constant, *T* is the absolute temperature,$\sigma$ is the Gaussian disorder width, and a conventional estimate value 2/3 for the coefficient $C$.

**Supplementary Note 6:** Time-resolved photoluminescence under different temperature of the Ternary (H) blend, Ternary (L) blend, D1:A1(1:1) blend (**b**), D1:D2 blend (**c**) and pure D1(**d**), Pump at 700nm. From room temperature to 70K, the lifetime of the exciton emission remains almost constant and there is no redshift emission tail in the first 20ps for the Ternary (H) blend. For the other two blends Ternary(L) and D1:A1 blend, there is a significant redshifted emission tail, and evolution with time and temperature. These results consistent with transient absorption data, demonstrate significant EET process in the D1:A1 and Ternary (L) blends.

**Supplementary References**

1. Tamai, Y. et al. Ultrafast Long-Range Charge Separation in Nonfullerene Organic Solar Cells. *ACS Nano* **11**, 12473-12481 (2017).

2. Shivanna, R. et al. Charge generation and transport in efficient organic bulk heterojunction solar cells with a perylene acceptor. *Energy. Environ. Sci.* **7**, 435-441 (2014).

3. Tvingstedt, K., Vandewal, K., Zhang, F., & Inganäs, O. On the Dissociation Efficiency of Charge Transfer Excitons and Frenkel Excitons in Organic Solar Cells: A Luminescence Quenching Study. *J. Phys. Chem. C* **114**, 21824-21832 (2010).

4. Vandewal, K. et al. Increased open-circuit voltage of organic solar cells by reduced donor-acceptor interface area. *Adv. Mater.* **26**, 3839-3843 (2014).

5. Sreearunothai, P. et al. Influence of Copolymer Interface Orientation on the Optical Emission of Polymeric Semiconductor Heterojunctions. *Phys. Rev. Lett.* **96**, 117403 (2006).

6. Damtie, F.A., Wacker, A., Pullerits, T. & Karki, K.J. Two-dimensional action spectroscopy of excitonic systems: Explicit simulation using a phase-modulation technique. *Phys. Rev. A* **96**, 053830 (2017).

7. Westenhoff, S. et al. Anomalous Energy Transfer Dynamics due to Torsional Relaxation in a Conjugated Polymer. *Phys. Rev. Lett.* **97**, 166804 (2006).

8. Yi, X. et al. Impact of Nonfullerene Molecular Architecture on Charge Generation, Transport, and Morphology in PTB7-Th-Based Organic Solar Cells. *Adv. Funct. Mater.* **28**, 1802702 (2018).

9. Lakowicz, J.R. Principles of Fluorescence Spectroscopy. *Springer Science & Business Media, New York.* (2013).

10. Gupta, V., Bharti, V., Kumar, M., Chand, S. & Heeger, A.J. Polymer-Polymer Forster Resonance Energy Transfer Significantly Boosts the Power Conversion Efficiency of Bulk-Heterojunction Solar Cells. *Adv. Mater.* **27**, 4398-4404 (2015).

11. Bi, P. et al. Dual Förster resonance energy transfer effects in non-fullerene ternary organic solar cells with the third component embedded in the donor and acceptor. *J. Mater. Chem. A. Mater* **5**, 12120-12130 (2017).

12. Vandewal, K., Tvingstedt, K., Gadisa, A., Inganäs, O. & Manca, J.V. Relating the open-circuit voltage to interface molecular properties of donor:acceptor bulk heterojunction solar cells. *Phys. Rev. B* **81**, 125204 (2010).

13. Baranovskii, S.D. Theoretical description of charge transport in disordered organic semiconductors. *Phys. Status. solidi (B)* **251**, 487-525 (2014).
