# Supplementary material for: Vibronic coherence contributes to photocurrent generation in organic semiconductor heterojunction diodes
Source: Nat Commun. 2020 Jan 30;11:617. doi: 10.1038/s41467-020-14476-w (PMC6992633; doi:10.1038/s41467-020-14476-w)
Supplement: Supplementary file 3 — Description of Additional Supplementary Files [file 41467_2020_14476_MOESM3_ESM.pdf]

Description of Additional Supplementary Files

File Name: Supplementary Movie 1
Description: in-plane breathing of the benzo[1,2-b;4,5-b']dithiophene donor unit

File Name: Supplementary Movie 2
Description: out-of-plane vibration of the 3-fluorothieno[3,4-b]thiophene donor unit

File Name: Supplementary Movie 3:
Description: out-of-plane vibration of the 3-fluorothieno[3,4-b]thiophene donor unit
